# Do video game interventions improve motor outcomes in children with developmental coordination disorder? A systematic review using the ICF framework

**DOI:** 10.1186/s12887-018-1381-7

**Published:** 2019-01-16

**Authors:** Benjamin F. Mentiplay, Tara L. FitzGerald, Ross A. Clark, Kelly J. Bower, Linda Denehy, Alicia J. Spittle

**Affiliations:** 1 Victorian Infant Brain Studies, Murdoch Children’s Research Institute, Melbourne, Australia; 2 La Trobe Sport and Exercise Medicine Research Centre, La Trobe University, Melbourne, Australia; 3 Department of Physiotherapy, University of Melbourne, Melbourne, Australia; 4 Faculty of Science, Health, Education and Engineering, University of the Sunshine Coast, Sippy Downs, Australia; 5 Neonatal Services, The Royal Women’s Hospital, Melbourne, Australia

**Keywords:** Virtual reality, Video games, Motor impairment, Developmental delay, Physiotherapy

## Abstract

**Background:**

Children with developmental coordination disorder (DCD) experience a range of difficulties that can potentially limit their academic, social and physical ability. Recent research has developed interventions that aim to improve motor outcomes in a variety of paediatric cohorts using video gaming equipment. Therefore, we aimed to systematically review the literature on virtual reality or video game interventions that aim to improve motor outcomes in children with DCD.

**Methods:**

Seven databases were searched for studies using the following criteria: a) virtual reality or video game based intervention; b) children with DCD; and c) motor outcomes relating to body structure and function, activity or participation. Data were extracted relating to study design, participant characteristics, details of the intervention, outcome measures, results, and feasibility/adherence.

**Results:**

Fifteen articles were included for review, including eight randomised controlled trials. No studies used virtual reality equipment, with all interventions using video games (Nintendo Wii in 12/15 articles). Mixed effects of video game intervention on outcome were found, with conflicting evidence across studies. Studies that reported on feasibility found most children enjoyed and adhered to the video game interventions.

**Conclusions:**

This review found limited evidence for the effectiveness of video game interventions for children with DCD to improve motor outcomes due to limitations in the research including low sample sizes and low to moderate methodological quality. Further research is needed to determine the effect of video game or virtual reality interventions on motor outcomes in children with DCD.

**Protocol registration:**

The protocol for this systematic review can be found on PROSPERO (CRD42017064427).

**Electronic supplementary material:**

The online version of this article (10.1186/s12887-018-1381-7) contains supplementary material, which is available to authorized users.

## Background

Developmental coordination disorder (DCD) is commonly reported to affect approximately 5 to 6% of the general school-aged population [1]. According to the Diagnostic and Statistical Manual for Mental Disorders, fifth edition (DSM-5) [2], DCD may be classified as a child meeting four criteria: a) motor coordination is below that expected for the child’s chronological age and intelligence level; b) the motor disorder interferes with academic achievement or activities of daily living; c) symptoms occur in the early developmental period; and d) the motor coordination is not due to a general medical condition (e.g. cerebral palsy). Children with DCD experience a range of difficulties in various domains (e.g. executive function, sensoriperceptual function, motor control of gait and posture) that can potentially limit their academic, social and physical ability [3, 4], as well as impacting upon their quality of life [5]. Previously it was thought that children with DCD would outgrow such difficulties, however evidence indicates that these impairments can continue later in life [6–9]. As such, importance is often placed on early interventions to improve motor difficulties in children with DCD.

A wide range of interventions have been used to improve motor impairment in children with DCD [10–14]. Emerging technology such as virtual reality and video game equipment has been the focus of recent research. The use of virtual reality or video game based interventions can provide a unique environment for children to improve, with potential benefits over more traditional methods including increased engagement, motivation, practice and repetition of movement as well as more challenging and varied activities and instantaneous visual and auditory feedback. Recent intervention studies have examined the effect of virtual reality or video game based interventions in a range of paediatric populations [15–22]. Due to the increasing volume of research, recent reviews have been performed to determine the effect of such interventions, however, the focus has primarily been on upper limb outcomes [16, 21] or on other paediatric populations such as cerebral palsy [15, 23, 24]. One recent review has examined the effectiveness of video games for improving motor outcomes in paediatric cohorts including DCD [25], however they examined multiple cohorts including cerebral palsy and Down syndrome and provided only a brief summary of the few results for children with DCD. The search strategy of this previous review was performed in 2015 [25] and with the constantly evolving nature of video game and virtual reality technology, further research studies may have been conducted in DCD. Further, the previous review did not examine the adherence to, or enjoyment of, video game intervention and did not determine if virtual reality equipment had been used in the DCD population.

Given that video gaming and virtual reality interventions are currently being used in paediatric rehabilitation with continually developing technology, and the possible benefits on improving motor outcomes for children with DCD, a further systematic review is warranted. Therefore, the aim of this review is to systematically collate and analyse the research that has used virtual reality or video game based interventions in children with DCD for the improvement of motor outcomes.

## Method

The systematic review followed the PRISMA guidelines [26] and the protocol can be found on PROSPERO (CRD42017064427). For this review, motor outcomes are described according to the International Classification of Functioning, Disability and Health, children and youth version (ICF-CY) framework [27]. The ICF-CY is a useful tool to understand the complex difficulties faced by children in three domains of body structure and function, activity, and participation. Body structures are defined as anatomical body parts and body functions are the physiological processes of the body, activity as the execution of a specific task or action and participation is broadly defined as involvement in a life situation [27]. The ICF-CY framework is used to holistically describe the impact of disability on individual functioning and on life experiences, with difficulties described as body structure and function impairments, activity limitations and participation restrictions.

### Search strategy

A systematic search of seven online databases (AMED, CINAHL, Cochrane, Embase, MEDLINE, Scopus, Web of Science) was conducted in July 2018 by one reviewer (author BFM). Key search terms and relevant synonyms were consistent across all databases, with relevant medical subject headings used where possible (see Additional file 1 for the search terms used). Multiple neurodevelopmental conditions were included in the systematic search to identify any articles that may have used a combined cohort that included children with DCD. No limitations were placed on publication date. Targeted searching was also performed of the reference lists of included articles to identify any additional studies not already found in the systematic database search.

### Selection criteria

Inclusion criteria involved: 1) children under the age of 18 years with DCD including a clear description of how DCD was defined; 2) an ‘immersive’ virtual reality (e.g. Oculus Rift) or video game (e.g. Nintendo Wii) based intervention of any research design such as randomised controlled trials (RCTs) or case studies; and 3) at least one outcome measure relating to motor and body structure and function impairment, activity limitation or participation restriction (e.g. mobility, gait, balance, strength, fitness, or physical activity).

Exclusion criteria were: 1) participants 18 years of age or older; 2) cohort of children with conditions other than DCD (e.g. acquired brain injury, cerebral palsy); 3) studies that did not provide a clear and consistent definition of DCD; 4) interventions that included robotics or assisted movement such as the Lokomat gait training device; 5) combined interventions where virtual reality or video games were not the main focus of the intervention program; 6) grey literature, review articles or conference abstracts; 7) full text articles not published in English; and 8) only outcome measures of upper limb function (e.g. reaching kinematics or the Melbourne Assessment of Unilateral Upper Limb Function [28]) or non-motor based measures (e.g. cognitive assessment).

### Selection of articles, data extraction and quality appraisal

Article selection, data extraction and quality assessment were completed independently by two reviewers (BFM and TLF) with a third reviewer (AJS) consulted for any discrepancies. The first step of article selection involved removing duplicates from the initial yield. The full texts of potential articles were then assessed by the two reviewers independently. The final articles to be included for review were agreed upon by all reviewers. A customised data extraction form was used to gather data including information on the study design, participant characteristics, details of the intervention, outcome measures, and results. Effect size (ES) and significant values were extracted only when clearly described. Data relating to the effect of intervention on typically developing (TD) children or the effect of variations of video game intervention were not extracted. Data were extracted relating to adherence to the intervention and any information about feasibility, enjoyment, or safety of intervention. Authors of included articles were contacted for further details if necessary.

Included articles were rated for methodological quality using the Downs and Black rating scale [29], which incorporates 27 questions that are appropriate for randomised and non-randomised intervention studies with questions relating to reporting, external validity, internal validity (bias and confounding), and power. Whilst this scale has shown good test-retest and inter-rater reliability, face and criterion validity [29], due to some ambiguity of question 27 of the scale [30, 31], this question was modified in accordance with previous research [32] to include a score of 0 or 1 depending on whether the study reported a power calculation (see Additional file 2 for the full scale). Interpretation of the overall quality of each study was based on previous research [33] and considered low if the study met < 60% of criteria on the scale, moderate for 60–74%, and high for ≥75%. Scores were based on the information within included articles, with additional information gained from related articles if necessary.

The level of evidence for each article was also classified using the hierarchy for interventional group designed studies from the American Academy of Cerebral Palsy and Developmental Medicine [34]. Studies were rated from the following: Level 1 evidence (large RCT, *n* > 100), Level 2 (smaller RCT, *n* < 100), Level 3 (cohort studies with concurrent control group), Level 4 (case series, cohort study without concurrent control group, or case-control study), or Level 5 evidence (case study or report, expert opinion, or anecdotes). As this review was interested in virtual reality and video game interventions in children with DCD, the level of evidence of each article was classified according to such interventions in children with DCD, irrespective of other methodological components included in the article (e.g. TD children).

## Results

**Fig. 1:**
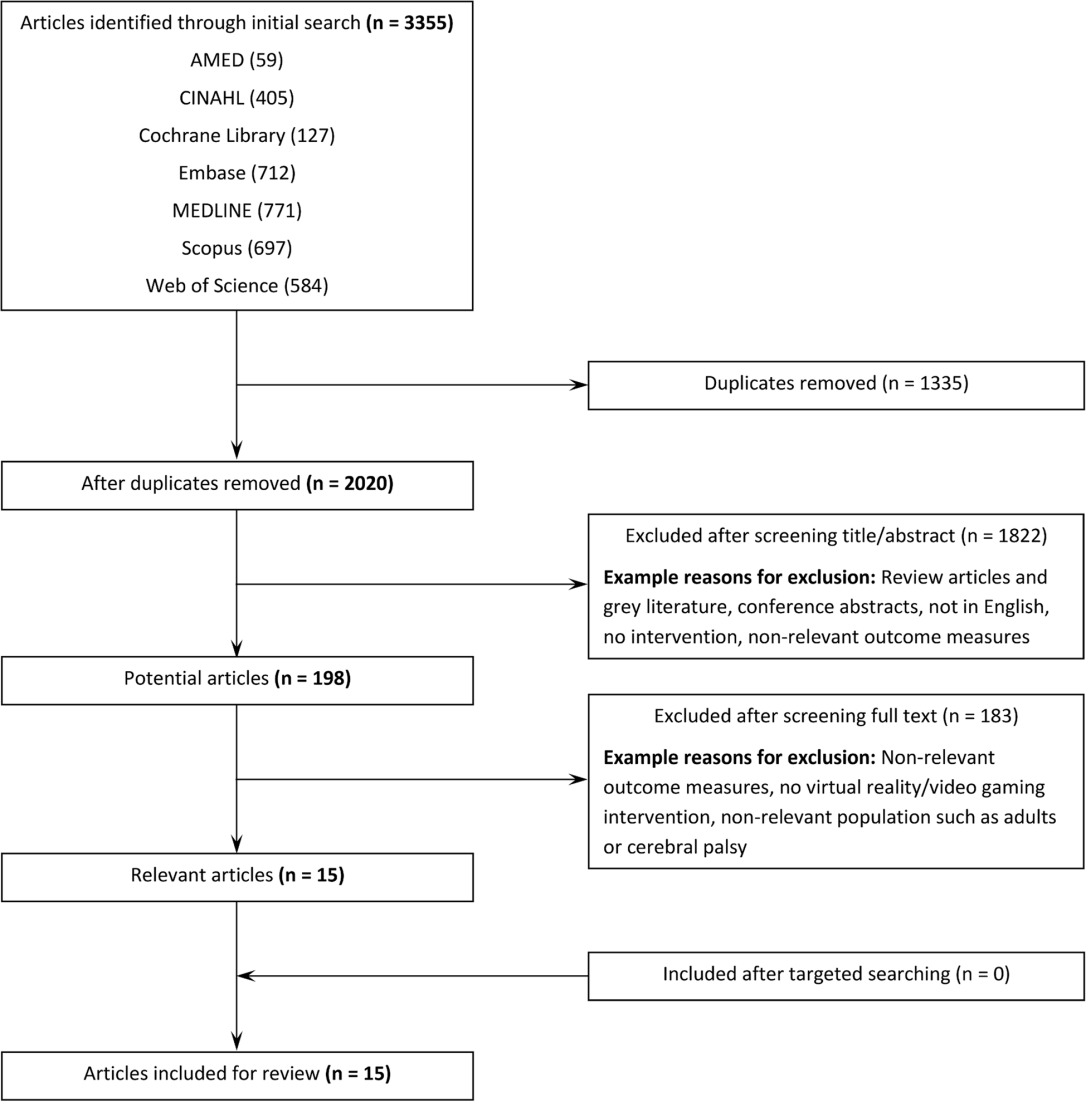
Flow diagram of search results

The steps involved in article selection are shown in Fig. 1, with 13 studies (total of 15 articles) identified as meeting the selection criteria [35–49]. There were instances of overlap between the cohorts and the interventions used, with the articles by Howie et al. [42] and Straker et al. [49] involving the same intervention and cohort (considered one study, but two articles). Two articles by Bonney et al. [37, 38] included the same interventions and cohort, and were also considered one study and two articles. The articles by Jelsma et al. [43, 44] included the same intervention and similar cohorts (only one intervention group used in the second study by Jelsma et al. [44]), the articles by Smits-Engelsman et al. [47, 48] involved the same intervention with different cohorts, and the other two articles by Bonney et al. [36, 39] also used the same intervention with different cohorts. As such these six articles were considered separate studies [36, 39, 43, 44, 47, 48].

### Study details

Table 1 includes details of the article characteristics. Overall sample sizes of children with DCD were relatively small ranging from 9 to 57, involving children aged 4 to 16 years. In total, 325 children with DCD (156 boys and 169 girls) and 101 TD children (53 boys and 48 girls) were involved in the interventions within studies. This total number of participants does not include the overlap of participants between articles.

**Table 1:** Study characteristics

Author	Sample Size	Research Design	Age, mean ± SD (range)	Gender, boys:girls	Control or comparison group?	Level of Evidence	Downs and Black
Ashkenazi et al. [35]	9 DCD	Non-randomised single group	5.6 ± 0.5 (4–6)	7:2	None	Level 4	16/27 (59%)
Bonney et al. [36]	43 DCD *21 (VG) 22 (comparison)*	RCT	VG: 14.3 ± 1.1 (13–16) Comparison: 14.4 ± 1.05 (13–16)	0:43	Comparison group: Task-oriented Functional Training (45mins, 1x week, 14 weeks)	Level 2	20/27 (74%)
Bonney et al. [37]	57 DCD 54 TD	RCT	DCD: 7.7 ± 1.0 (6–10) TD: 7.6 ± 1.0 (6–10)	DCD: 29:28 TD: 28:26	Two DCD groups for variable and repetitive video game practice (also comparison to TD)	Level 2	21/27 (78%)
Bonney et al. [38]	57 DCD 54 TD	RCT	DCD: 7.7 ± 1.0 (6–10) TD: 7.6 ± 1.0 (6–10)	DCD: 29:28 TD: 28:26	Two DCD groups for variable and repetitive video game practice (also comparison to TD)	Level 2	20/27 (74%)
Bonney et al. [39]	16 DCD	Non-randomised single group	14.5 ± 1.0 (13–16)	0:16	None	Level 4	19/27 (70%)
Ferguson et al. [40]	46 DCD *19 (VG)* *27 (comparison)*	Non-randomised with comparison group	VG: 7.6 ± 1.1 (6–10) Comparison: 8.2 ± 1.3 (6–10)	VG: 9:10 Comparison: 15:12	Comparison group: NeuroMotor Task Training (45–60 min, 2x week, 9 weeks)^a^	Level 3	18/27 (67%)
Hammond et al. [41]	18 DCD *10 (group A)* *8 (group B)*	Crossover RCT	Group A: 8.5 ± 1.2 (7.1–10.7) Group B: 9.5 ± 1.4 (7.2–10.9)	Group A: 8:2 Group B: 6:2	Crossover design with school-run motor skills program (1 h, 1x week, 4 weeks)^a^	Level 2	17/27 (63%)
Howie et al. [42]	21 DCD *11 (group A)* *10 (group B)*	Crossover RCT	11 ± 1.0 (10–12)	10:11	Crossover design with no intervention and avoidance of active video gaming	Level 2	18/27 (67%)
Jelsma et al. [43]	28 DCD (20 TD^b^) *14 (group A)* *14 (group B)*	Cohort study with intervention	8.2 ± 1.4 (5.9–11.3)	18:10	Group A: 6 weeks of intervention. Group B: 6 weeks of no intervention then 6 weeks of intervention	Level 3	15/27 (56%)
Jelsma et al. [44]	14 DCD^c^ (20 TD^b^)	Cohort study with self-control intervention	7.7 ± 1.2 (5.9–9.5)	9:5	DCD group: 6 weeks of no intervention followed by 6 weeks of intervention (same as Group B in Jelsma et al. 2014 [43])	Level 4	15/27 (56%)
Ju et al. [45]	24 DCD (12 TD) *12 (VG)* *12 (DCD control)* *12 (TD control)*	RCT	DCD VG: 6.8 ± 1.3 (5–10) DCD control: 7.0 ± 1.5 (5–10) TD control: 7.3 ± 1.6 (5–10)	DCD VG: 6:6 DCD control: 7:5 TD control: 7:5	DCD and TD control groups had no intervention	Level 2	14/27 (52%)
Mombarg et al. [46]	29 DCD *15 (VG)* *14 (control)*	RCT	VG: 9.5 ± 1.8 (7–12) Control: 9.7 ± 1.1 (7–12)	VG: 12:3 Control: 11:3	Control group had no intervention	Level 2	17/27 (63%)
Smits-Engelsman et al. [48]	17 DCD 17 TD	Cohort study with intervention	DCD: 7.9 ± 1.2 (6–10) TD: 7.7 ± 1.1 (6–10)	DCD: 9:8 TD: 9:8	Comparison group of TD with same intervention	Level 3	17/27 (63%)
Smits-Engelsman et al. [47]	17 DCD 18 TD	Cohort study with intervention	DCD: 8.2 ± 1.1 (6–10) TD: 8.0 ± 1.2 (6–10)	DCD: 9:8 TD: 9:9	Comparison group of TD with same intervention	Level 3	18/27 (67%)
Straker et al. [49]	21 DCD *11 (group A)* *10 (group B)*	Crossover RCT	11 ± 1.0 (10–12)	10:11	Crossover design with no intervention and avoidance of active video gaming	Level 2	18/27 (67%)

*SD* standard deviation, *DCD* developmental coordination disorder, *TD* typically developing children, *VG* video game group, *RCT* randomised controlled trial; ^a^different duration/frequency to video game intervention; ^b^typically developing children for baseline comparisons only; ^c^14 participants performed the intervention while 28 participants with DCD were included overall

Table 2 provides information on the interventions included in each article. All articles used video gaming equipment for the interventions, with the Nintendo Wii being the most commonly used device in 12/15 articles (11/12 articles used the Wii Fit gaming software) [36–41, 43–48]. The other three articles included the PlayStation2 EyeToy [35], or a combination of the PlayStation3 Move and Eye with the Xbox 360 Kinect [42, 49]. All of these video games are considered ‘active’ as they require various forms of participant movement in contrast with traditional sedentary video games. No study used any ‘immersive’ virtual reality devices. Session durations ranged from 10 to 45 min of video gaming, with interventions lasting from 4 to 16 weeks. The setting for intervention was either at school [36–41, 43–48] or home-based [42, 49], with the intervention setting unclear in one article [35].

**Table 2:** Details of the video game interventions in included articles

Author	VG equipment	Session duration	Frequency	VG intervention duration	VG intervention setting
Ashkenazi et al. [35]	PlayStation2 EyeToy^a^	60mins (45mins video games)^b^	1x week (total of 10 sessions)	12 weeks	Unclear
Bonney et al. [36]	Nintendo Wii^d^	45mins	1x week	14 weeks	School
Bonney et al. [37]	Nintendo Wii	20mins	2x week	5 weeks	School
Bonney et al. [38]	Nintendo Wii	20mins	2x week	5 weeks	School
Bonney et al. [39]	Nintendo Wii^d^	45mins	1x week	14 weeks	School
Ferguson et al. [40]	Nintendo Wii	30mins	3x week	6 weeks	School
Hammond et al. [41]	Nintendo Wii	10mins	3x week	4 weeks	School
Howie et al. [42]	PlayStation3 Move and Eye, Xbox 360 Kinect	Minimum of 20mins	Most days (minimum of 4–5 days)	16 weeks	Home-based
Jelsma et al. [43]	Nintendo Wii	30mins	3x week	6 weeks	School^c^
Jelsma et al. [44]	Nintendo Wii	30mins	3x week	6 weeks	School^c^
Ju et al. [45]	Nintendo Wii^e^	45mins	3x week	4 weeks	School^c^
Mombarg et al. [46]	Nintendo Wii	30mins	3x week	6 weeks	School
Smits-Engelsman et al. [48]	Nintendo Wii	20mins	2x week	5 weeks	School
Smits-Engelsman et al. [47]	Nintendo Wii	20mins	2x week	5 weeks	School
Straker et al. [49]	PlayStation3 Move and Eye, Xbox 360 Kinect	Minimum of 20mins	Most days (minimum of 4–5 days)	16 weeks	Home-based

VG, video game; ^a^some games played with parents, some on different surfaces to increase instability; ^b^last 15 min of session was for goal-directed tasks (e.g. riding a bicycle) and as such video games were only played for 45 min per session; ^c^determined after contacting author; ^d^intervention with the Nintendo Wii also included weighted backpacks to augment body mass and a wooden platform to raise the centre of mass; ^e^used custom-made games that integrated with the Nintendo Wii

### Quality appraisal

The level of evidence varied across the included articles with eight categorised as Level 2 [36–38, 41, 42, 45, 46, 49], four as Level 3 [40, 43, 47, 48], and three as Level 4 [35, 39, 44]. The majority of articles had moderate overall quality (60–74%) [36, 38–42, 46–49] as assessed by the Downs and Black scale, with four articles demonstrating low overall quality (< 60%) [35, 43–45]. Only one article received a high total quality score (≥75%) [37] (see Additional file 3 for results). Overall, articles described their aims, outcome measures, participant characteristics and the main findings of the study well, however only one article described adverse events, with no injuries reported during the intervention [39]. It is acknowledged that it is difficult to blind participants to video gaming interventions and concealed assignment is similarly difficult to implement. Seven articles provided a sample size calculation [36–40, 42, 49].

### Outcome measures

Outcome measures were varied across articles. The most common outcome measure was the Movement Assessment Battery for Children, second edition (MABC-2), with nine articles using the MABC-2 total score or sub-scores [35, 36, 38, 40, 43, 45, 46, 48, 49]. The Bruininks Oseretsky Test of Motor Proficiency, second edition (BOT-2) was used in seven articles [36, 38, 41, 43, 46–48]. Various other outcomes were used including but not limited to accelerometer measured physical activity [42], muscle strength [36, 38, 40, 47], anaerobic performance [36, 38, 40, 47], and the developmental coordination disorder questionnaire (DCD-Q) [35, 49].

Due to the heterogeneity among articles and the limited number of Level 2 studies, we were unable to complete a meta-analysis. The outcomes were summarised using the ICF-CY framework and collated within the levels of evidence of included articles.

### Outcomes of level 2 and 3 studies

A variety of study designs and outcome measures were included in the Level 2 and 3 articles.

#### Body structure and function

Four Level 2 articles [36, 38, 45, 49] and two Level 3 articles [40, 47] used outcomes relating to body structure and function. The results of these outcomes relat/./ing to body structure and function are detailed in Table 3, which include strength, anaerobic performance, aerobic fitness and static and dynamic balance.

**Table 3:** Body structure and function outcomes after video game intervention

Author	Control or Comparison group		Experimental group		Outcome measure	Experiment improvement?	Effect over control?
	N	Intervention	N	Video game intervention			
***Level 2: Strength***							
Bonney et al. [36]	22	Task-oriented Training	21	Nintendo Wii	1 FSM item	✔	No diff
					Isometric Strength^d^	✔	No diff
Bonney et al. [38]^e^	–	–	57	Nintendo Wii	FSM items	✔	N/A
***Level 3: Strength***							
Ferguson et al. [40]	27	Neuromotor training	19	Nintendo Wii	FSM total score	✖	✖^a^
					FSM items	✔^b^	✖^a^
					Isometric Strength^d^	✖	✖
Smits-Engelsman et al. [47]^e^	–	–	17	Nintendo Wii	FSM lower limb items	✔	N/A
***Level 2: Anaerobic performance***							
Bonney et al. [36]	22	Task-oriented Training	21	Nintendo Wii	10x5m sprints	✔	No diff
					10x5m slalom sprints	✔	No diff
Bonney et al. [38]^e^	–	–	57	Nintendo Wii	10x5m sprints	✖	N/A
					10x5m slalom sprints	✔	N/A
***Level 3: Anaerobic performance***							
Ferguson et al. [40]	27	Neuromotor training	19	Nintendo Wii	Muscle Power Sprint Test	✔	✖^a^
Smits-Engelsman et al. [47]^e^	–	–	17	Nintendo Wii	10x5m sprints	✔	N/A
					10x5m slalom sprints	✔	N/A
***Level 2: Aerobic fitness***							
Bonney et al. [36]	22	Task-oriented Training	21	Nintendo Wii	20 m shuttle run test	✖	✖
***Level 3: Aerobic fitness***							
Ferguson et al. [40]	27	Neuromotor training	19	Nintendo Wii	20 m shuttle run test	✖	✖^a^
***Level 2: Static and dynamic balance (force plate)***							
Ju et al. [45]	12	No intervention	12	Nintendo Wii	Static force plate measures	✔^c^	✔^c^
					Dynamic force plate measures	✔	✔
Straker et al. [49]	21	No intervention	21	PlayStation3 Move and Eye	Static force plate measures	NR	No diff
				Xbox 360 Kinect			

✔, significant improvement/effect; ✖, no significant improvement/effect; ✖^a^, control/comparison group had significantly greater improvements; No diff, no difference between experimental and comparison/control group; N/A, not applicable due to study design; NR, not reported in article; FSM, Functional Strength Measure; ^b^one item out of eight (lifting a box) showed significant improvement after experimental intervention; ^c^duration of single leg standing showed significant experimental improvements and effect over control, but no improvements were shown for the centre of pressure trajectory in either group; ^d^isometric strength taken from the knee extensors, ankle plantarflexors and ankle dorsiflexors by Bonney et al. [36] and from the elbow flexors, elbow extensors, knee extensors and grip strength by Ferguson et al. [40]; ^e^control/comparison group included typically developing children or variation of video game intervention

##### Strength

Strength was measured in four studies with the Functional Strength Measure (FSM) [50]. Bonney et al. [36] showed children with DCD significantly improved in the one item of the FSM that was measured (stair climbing; ES = − 0.79), with similar improvements shown in the comparison task-oriented training group (ES = − 0.57). Another study by Bonney et al. [38] showed that both groups of children with DCD (variable and repetitive video game training groups) had significant and similar improvements in items of the FSM (*p* < 0.001). Ferguson et al. [40] showed that a comparison group of neuromotor task training had significantly greater improvements on the total score and all eight items of the FSM compared with the video game intervention. The video game intervention group only had significant strength improvements on one item of the FSM (lifting a box; *p* = 0.01; ES = − 0.58) [40]. Conversely, Smits-Engelsman et al. [47] showed that video game intervention significantly improved FSM scores (only lower limb items were assessed) in children with DCD (*p* < 0.05), with large ESs seen across the lower limb items (ES = − 0.8 to − 3.9). Isometric strength was also measured with hand-held dynamometry in two studies [36, 40]. Bonney et al. [36] showed significant improvements and large ESs in isometric strength (ES = − 2.84 to − 4.15) that was similar to the comparison group (ES = − 3.12 to − 6.64), whereas Ferguson et al. [40] showed no significant improvements in isometric strength in either the comparison group or the video game intervention group (*p* > 0.05) [40].

##### Anaerobic performance

Anaerobic performance was assessed using the Muscle Power Sprint Test [51] by Ferguson et al. [40] whereas the two studies by Bonney et al. [36, 38] as well as Smits-Engelsman et al. [47] used a protocol measuring the time taken to complete: (1) 10 by 5 m straight sprints; and (2) 10 by 5 m slalom sprints. Results for the Muscle Power Sprint Test showed significant improvements after video game intervention (*p* = 0.01; ES = − 0.56), although the neuromotor task training comparison group had greater improvements [40]. Bonney et al. [36] found significant improvements in both sprint tests (*p* = 0.001; ES = 1.14 to 1.32) with similar yet slightly smaller improvements shown in the task-oriented comparison group (*p* = 0.002; ES = 0.58 to 0.75). Interestingly, the other article by Bonney et al. [38] showed significant improvements in the slalom sprints in children with DCD (*p* < 0.001), although no improvements were shown for the straight sprints (*p* = 0.075). Smits-Engelsman et al. [47] showed a similar trend with a moderate ES for the straight sprints (ES = 0.7) and a large ES shown for the slalom sprints (ES = 2.2), with both sprints showing significant improvement in children with DCD (*p* < 0.05).

##### Aerobic fitness

Aerobic fitness was assessed using the 20 m shuttle run test by Bonney et al. [36] and Ferguson et al. [40]. The video game intervention in both studies did not result in significant improvements in aerobic fitness. Interestingly, the task-oriented comparison group in Bonney et al. [36] did not significantly improve aerobic fitness, whereas the neuromotor task training comparison group in Ferguson et al. [40] had a significant improvement after intervention (*p* = 0.02; ES = − 1.15).

##### Static and dynamic balance

Ju et al. [45] examined static and dynamic tasks that replicated the custom-made Nintendo Wii games from their intervention group. This study showed significant improvements for the static task in the intervention group for the duration of a single leg stance over the control group (no intervention), but not for the centre of pressure trajectory. Analysis of the dynamic task (successful trials and centre of pressure trajectory) showed significant improvements for the intervention group over the control group [45].

Straker et al. [49] examined static balance with a single leg balance task where children were asked to stand on their preferred leg for as long as possible, while a force platform recorded various centre of mass measures. All measures of static balance showed no significant differences between the video game intervention and control phases (*p* = 0.300 to 0.559).

#### Activity

Activity domain outcome measures were reported in six Level 2 [36, 38, 41, 45, 46, 49] and four Level 3 articles [40, 43, 47, 48]. The results of these articles are shown in Table 4. Outcome measures included the MABC-2, BOT-2, DCD-Q, and child reported questionnaires of motor skills. Video game performance was also assessed in four articles [37, 43, 48, 49], although as video game performance has limited relevance to functional activities of daily living these results were not included in Table 4.

**Table 4:** Activity outcomes after video game intervention

Author	Control or Comparison group		Experimental group		Outcome measure	Experiment improvement?	Effect over control?
	N	Intervention	*N*	Video game intervention			
***Level 2: MABC-2***							
Bonney et al. [36]	22	Task-oriented Training	21	Nintendo Wii	Total score	✔	No diff
					Manual dexterity sub-score	✔	No diff
					Balance sub-score	✔	No diff
					Aiming and catching sub-score	✖	✖
Bonney et al. [38]^e^	–	–	57	Nintendo Wii	Total score	✔	N/A
					Manual dexterity sub-score	✔	N/A
					Balance sub-score	✔	N/A
					Balance item	✔	N/A
					Aiming and catching sub-score	✔	N/A
Ju et al. [45]	12	No intervention	12	Nintendo Wii	Balance sub-score	✔	✔
Mombarg et al. [46]	14	No intervention	15	Nintendo Wii	Balance sub-score	✔	✔
					Balance items	✔^c^	No diff
Straker et al. [49]	21	No intervention	21	PlayStation3 Move and Eye	Total score	NR	No diff
				Xbox 360 Kinect	Manual dexterity sub-score	NR	No diff
					Balance sub-score	NR	No diff
					Aiming and catching sub-score	NR	No diff
***Level 3: MABC-2***							
Ferguson et al. [40]	27	Neuromotor training	19	Nintendo Wii	Total score	✖	✖^a^
					Manual dexterity sub-score	✖	✖^a^
					Balance sub-score	✖	✖^a^
					Aiming and catching sub-score	✖	No diff
Jelsma et al. [43]	14	No intervention	28^b^	Nintendo Wii	Total score	✔	✔
					Manual dexterity sub-score	✖	NR
					Balance sub-score	✔	✔
					Aiming and catching sub-score	✖	NR
Smits-Engelsman et al. [48]^e^	–	–	17	Nintendo Wii	Balance items	✔	N/A
***Level 2: BOT-2***							
Bonney et al. [36]	22	Task-oriented Training	21	Nintendo Wii	Running and agility sub-score	✔	No diff
Bonney et al. [38]^e^	–	–	57	Nintendo Wii	Balance sub-score	✔	N/A
					Running and agility sub-score	✔	N/A
Hammond et al. [41]	18	School-run motor skills	18	Nintendo Wii	Total score	✔	✔
					Fine precision sub-score	NR	NR
					Fine integration sub-score	NR	NR
					Manual dexterity sub-score	NR	NR
					Coordination sub-score	NR	NR
					Balance sub-score	NR	NR
					Running and agility sub-score	NR	NR
					Upper coordination sub-score	NR	NR
					Strength sub-score	NR	NR
Mombarg et al. [46]	14	No intervention	15	Nintendo Wii	Balance sub-score	✔	✔
					Balance items	✔^d^	✔^d^
					Running and agility sub-score	✔	No diff
					Running and agility items	NR	NR
***Level 3: BOT-2***							
Jelsma et al. [43]	14	No intervention	28^b^	Nintendo Wii	Coordination sub-score	✔	✔
					Balance sub-score	✔	No diff
					Running and agility sub-score	✔	✔
Smits-Engelsman et al. [48]^e^	–	–	17	Nintendo Wii	Balance item	✔	N/A
Smits-Engelsman et al. [47]^e^	–	–	17	Nintendo Wii	Balance sub-score	✔	N/A
					Running and agility sub-score	✖	N/A
***Level 2: DCD-Q***							
Straker et al. [49]	21	No intervention	21	PlayStation3 Move and Eye	Total score	✖	No diff
				Xbox 360 Kinect			
***Level 2: Child reported questionnaires***							
Hammond et al. [41]	18	School-run motor skills	18	Nintendo Wii	CSQ Ability score	✔	No diff
					CSQ Satisfaction score	✔	No diff
Straker et al. [49]	21	No intervention	21	PlayStation3 Move and Eye	Perceived physical skills	NR	✔
				Xbox 360 Kinect			

✔, significant improvement/effect; ✖, no significant improvement/effect; ✖^a^, control/comparison group had significantly greater improvements; No diff, no difference between experimental and comparison/control group; N/A, not applicable due to study design; NR, not reported in article; MABC-2, Movement Assessment Battery for Children, second edition; BOT-2, Bruininks Oseretsky Test of Motor Proficiency, second edition; DCD-Q, Developmental Coordination Disorder Questionnaire; CSQ, Co-ordination Skills Questionnaire; ^b^28 participants completed experimental intervention, 14 were used for comparison to control group; ^c^two out of three items (walking on a line, jumping) showed significant improvement after experimental intervention; ^d^one out of nine items (standing on one leg on a balance beam) showed significant improvement after experimental intervention, with this improvement greater than control/comparison; ^e^control/comparison group included typically developing children or variation of video game intervention

##### Movement assessment battery for children

Five Level 2 articles used the MABC-2 [36, 38, 45, 46, 49]. Bonney et al. [36] showed significant improvements in the total standard score of the MABC-2 as well as the manual dexterity and balance sub-scores (ES = − 0.95 to − 2.53), with similar improvements shown in their comparison group (ES = − 0.69 to − 1.41). Both groups showed no improvement in the aiming and catching sub-score of the MABC-2 [36]. The other article by Bonney et al. [38] showed significant improvements for children with DCD following video game intervention in the MABC-2 total score, manual dexterity sub-score, balance sub-score (plus one balance item), and aiming and catching sub-score (*p* < 0.01) [38]. Ju et al. [45] showed significant improvement in the balance sub-score of the MABC-2 in the intervention group, with no improvement observed in the control group (no intervention). Mombarg et al. [46] found the video game intervention group significantly improved on the balance sub-score of the MABC-2 with this improvement greater than the control group. Secondary analysis of the balance sub-score items showed that two out of three balance items significantly improved after video game intervention, although no difference was shown with the control group [46]. Straker et al. [49] found no significant difference between a video game intervention phase and a control phase for the MABC-2 total score, manual dexterity sub-score, balance sub-score, and aiming and catching sub-score.

Of the Level 3 articles that used the MABC-2, Ferguson et al. [40] found that the video game intervention did not statistically improve any component of the MABC-2 (*p* = 0.08 to 0.87). The comparison group (neuromotor task training) showed significantly larger improvements in MABC-2 total score, balance sub-score, and manual dexterity sub-score compared with the video game intervention. There were no significant improvements on the aiming and catching sub-score of the MABC-2 for either group [40]. Jelsma et al. [43] included two groups; one completed video game intervention, and a second group completed a control phase of no intervention followed by video game intervention. This article found significant improvements in the total score and balance sub-score (*p* < 0.01), with these improvements significantly greater than the control phase. However, the manual dexterity and aiming and catching sub-scores of the MABC-2 showed no improvements (*p* > 0.05) [43]. Smits-Engelsman et al. [48] examined the effect of video game intervention in a group of children with DCD and TD children. This article found significant improvements in five items of the balance sub-score of the MABC-2 after video game intervention in children with DCD (*p* < 0.05) [48].

##### Bruininks Oseretsky test of motor proficiency

Four Level 2 articles [36, 38, 41, 46] reported outcomes with the BOT-2. Bonney et al. [36] only examined the running and agility sub-score of the BOT-2 and found significant improvements after video game intervention (ES = − 1.75), with similar improvements found in the comparison group (ES = − 1.23). The other article by Bonney et al. [38] showed significant improvements in children with DCD in the balance sub-score and running and agility sub-score of the BOT-2 (*p* < 0.001). Using a crossover RCT with a comparison phase (school-run motor skills program) and an experimental phase of video game intervention, Hammond et al. [41] found that the total score of the BOT-2 significantly improved after video game intervention, with this improvement greater than the comparison intervention. Hammond et al. [41] did not perform any statistical analyses for each of the eight sub-scores of the BOT-2. Mombarg et al. [46] examined two sub-scores of the BOT-2, balance as well as running speed and agility, and presented results for the individual items of these two sub-scores. The video game intervention group significantly improved within the balance sub-score, with the improvement greater than the control group [46]. The article also showed that one balance item out of the nine assessed showed significant improvements after video game intervention (standing on one leg on a balance beam), with this one item showing greater improvements over the control group. Mombarg [46] found that both the control group and video game intervention group improved on the BOT-2 running speed and agility sub-score, with no difference between groups. The results for the individual items of the running speed and agility sub-score were not clearly reported.

Of the Level 3 articles that used the BOT-2, Jelsma et al. [43] found significant improvements after video game intervention for three BOT-2 sub-scores (bilateral coordination, balance, and running speed and agility; *p* < 0.001). With the exception of the balance sub-score, these were significantly greater improvements compared with the control phase [43]. The two articles by Smits-Engelsman et al. [47, 48] examined the effect of video game intervention in groups of children with DCD and TD children. Smits-Engelsman et al. [48] examined just one item of the BOT-2 balance sub-score (single leg stance on a balance beam) and found children with DCD significantly improved after video game intervention (*p* = 0.042). Smits-Engelsman et al. [47] examined two sub-scores of the BOT-2 (balance, and running speed and agility) and found significant improvements only in the balance sub-score in children with DCD (*p* = 0.003).

##### Developmental coordination disorder questionnaire

Straker et al. [49] (Level 2) used the DCD-Q total score and found no significant improvement following video game intervention, with no difference between the control and video game phases (*p* = 0.082).

##### Child reported questionnaires

Two articles (both classified as Level 2) used child reported questionnaires of motor skills [41, 49]. Hammond et al. [41] used the Co-ordination Skills Questionnaire [52] in a crossover RCT design, with a comparison intervention phase. Children’s perception of their motor ability and satisfaction significantly improved over the intervention period, although there was no difference with the comparison intervention [41]. Straker et al. [49] used a global change score of child reported motor coordination, using one question and an 11-point Likert type scale: ‘compared to when you started the study, how would you describe your physical skills at the end of the active games block?’. The video game intervention resulted in improved perceived physical skills that was significantly higher than the control phase (*p* < 0.001) [49].

##### Video game performance

Four articles examined changes after intervention in performance on specific video games [37, 43, 48, 49]. Straker et al. [49] (Level 2) found no significant difference in video game performance (four various games) between the control phase and the video game phase (*p* = 0.29 to 0.93). In contrast, Bonney et al. [37] (Level 2) showed children with DCD improved video game performance after intervention in two groups performing either repetitive training (same Wii game) or variable training (various Wii games). Two Level 3 studies found improvements in video game performance after intervention [43, 48], although one of these studies showed the improvements in video game performance after intervention was not significantly higher than improvements seen after a control phase [43].

#### Participation

Only two articles (both classified as Level 2) examined outcomes related to participation [36, 42]. Howie et al. [42] used a crossover RCT design to examine habitual physical activity measures taken from accelerometry, with phases of either no intervention or video game intervention. A range of habitual physical activity outcomes were collected during one week of wearing an accelerometer. There were no significant differences in any physical activity outcomes between the 16 week intervention or control phases (*p* = 0.249 to 0.865). Bonney et al. [36] examined two questionnaires related to participation; the Children’s Self-Perceptions of Adequacy in and Predilection for Physical Activity (CSAPPA), and a questionnaire developed by the authors specifically for their study to examine change following the intervention period, the Participation in Activities of Daily Living for Adolescents Questionnaire (PADLA-Q). The results showed improvements after intervention for the total CSAPPA score and the predilection sub-score, but not for the adequacy or enjoyment sub-scores; whilst there was no difference between the video game and comparison interventions on the PADLA-Q [36].

### Outcomes of level 4 studies

Three articles were classified as Level 4 [35, 39, 44], with these articles reported in the text only. The Level 4 studies included a single group design.

#### Body structure and function

Ashkenazi et al. [35] examined walking tasks pre and post intervention (only 5/9 participants completed the walking assessments). The walking tasks included: (1) the 6 min walk test; (2) 10 m walk test; (3) 10 m walk test while carrying a tray with a cup two thirds filled with water; and (4) 10 m walk test while carrying the same tray and talking by answering questions. No significant improvements were found after video game intervention (*p* = 0.138 to 0.715), with small to medium ESs reported (ES = 0.10 to 0.74) [35].

Bonney et al. [39] included a single group of 16 girls with probable DCD and examined the 6 min walk test (as a measure of aerobic fitness) and the Muscle Power Sprint Test (as a measure of anaerobic performance). This article showed a significant improvement in the 6 min walk test and the Muscle Power Sprint Test [39].

Jelsma et al. [44] included a single group, who were the second group of the first publication by Jelsma et al. [43]. This article examined dynamic balance measures with a force plate during Wii game play [44]. Force plate measures of balance significantly improved after the initial control phase, although no further significant change was found after the video game intervention, suggesting that video game intervention did not improve dynamic balance during Wii game play.

#### Activity

Ashkenazi et al. [35] examined outcomes from the MABC-2 and the DCD-Q (with data from all 9 participants). Significant improvements were reported for the MABC-2 total score (*p* = 0.024; ES = 0.93) and the balance sub-score (*p* = 0.012; ES = 1.26), whilst improvements that did not reach significance were shown for the manual dexterity (*p* = 0.088; ES = 0.61) and aiming and catching sub-scores (*p* = 0.44; ES = 0.23). Significant improvements were shown for the DCD-Q total score (*p* = 0.05; ES = 0.68) and the movement control sub-score (*p* = 0.036; ES = 0.88), with smaller non-significant improvements for the fine motor (*p* = 0.123; ES = 0.55) and coordination sub-scores (*p* = 0.128; ES = 0.53) [35].

#### Participation

No outcomes were reported for participation in Level 4 articles.

### Adherence, enjoyment and safety

Eight out of 13 studies reported adherence to the intervention [36–38, 40, 42, 43, 46–49]. Adherence ranged from 92 to 100% for sessions attended in seven studies [36–38, 40, 43, 46–48], while one study reported that 90% of participants met the minimum requirements of video game play per week (80 min) [42, 49]. Five studies [37, 39, 43, 47, 48] reported child enjoyment by using a 5-point enjoyment scale, with responses as either: no fun at all; boring; a bit of fun; fun; super fun (or awesome). At the end of the interventions, 86 to 100% of children reported the video games to be either ‘fun’ or ‘super fun/awesome’. Another study reported parental satisfaction after the intervention [35], with eight out of nine parents reporting high satisfaction with the video game intervention. One other study reported the experiences of the training supervisors [39]. Three studies did not report any outcome related to adherence, enjoyment or feasibility [41, 44, 45]. Only one article reported on adverse events, with this article by Bonney et al. [39] reporting no injuries during the intervention.

## Discussion

This systematic review collated intervention based research examining the effectiveness of video game or virtual reality technology for improving motor outcomes in children with DCD. The limited number of articles with varying levels of evidence, research design, and sample sizes made comparison between studies difficult. The ICF-CY framework was used to classify outcomes, with mixed intervention effects. Limited evidence suggests that video game intervention may have some benefits for body structure and function impairments and activity limitations, although studies were conflicted in their findings and often found similar results to control or comparison groups, with one article showing better results with a comparison intervention [40]. Only one article reported on participation restrictions, with no significant effect of video game intervention found over no intervention [42]. Studies reported that the majority of children enjoyed and adhered to the video game interventions. Further studies with large sample sizes and rigorous methodological design are needed to determine the effectiveness of video game interventions on motor impairment in children with DCD.

The use of video game based interventions in paediatric rehabilitation has become more prevalent in recent research [15, 16, 24]. The technology used in these interventions is constantly evolving, making up to date systematic reviews imperative. However, due to this constant technological development, direct comparisons between studies is problematic. Compounding this problem is the limited research that has been undertaken, with only 15 articles included in this review. The 15 articles in the current review had limitations in study design (only 8/15 were RCTs), relatively small sample sizes of children with DCD (*n* = 9 to 57), and most articles had low to moderate methodological quality with only one article scored as high [37] (scores ranging from 14 to 21 out a maximum score of 27). Due to these limitations and the heterogeneity in outcome measures between studies, a meta-analysis was unable to be performed to statistically compare studies. As such, future research is needed to comprehensively determine the effectiveness of video game based interventions on motor impairment and physical function in children with DCD.

The included studies showed mixed results within the body structure and function domain. Video game intervention showed varied results for strength outcomes [36, 38, 40, 47], significant improvements for anaerobic performance [36, 38, 40, 47] and no improvements in aerobic fitness [36, 40]. Interestingly, neuromotor task training provided significantly greater improvements in strength, anaerobic performance and aerobic fitness compared to the video game intervention in one study [40]. Whilst these results may suggest that task specific training is important, this finding of greater improvements over the video game intervention was only from one Level 3 article [40]. Additionally, similar improvements in strength, anaerobic performance and aerobic fitness were found between the video game intervention and a task-oriented functional training intervention in one Level 2 article [36]. Video game intervention also showed mixed results for walking outcomes and static and dynamic balance measures taken from a force plate [35, 39, 44, 45, 49]. Overall, it appears that although video game intervention may provide some improvement in the body structure and function domain, the improvements were often not significant and similar to comparison interventions.

Activity domain outcomes were the most commonly assessed across the studies. Some articles showed video game intervention to significantly improve MABC-2 outcomes [36, 38, 43, 45, 46, 48], with mixed results shown for video game interventions in comparison with a control group or comparison interventions. Outcomes for the BOT-2 appeared to consistently show improvements after video game intervention in six articles [36, 38, 41, 43, 46–48]. Taken in isolation from other outcomes, the results of the BOT-2 suggest that video game intervention improves the activity domain in children with DCD, however other outcomes need to be considered. Other outcomes relating to the activity domain were the DCD-Q [35, 49] and child reported questionnaires [41, 49]. Improvements in the DCD-Q scores after video game intervention were shown in one Level 4 article [35], with no difference between control and video game intervention shown in a Level 2 article [49]. Child report questionnaires after video game intervention showed improvements [41, 49], suggesting that the perceived motor skills ability of children with DCD improves after intervention. Due to the mixed results between studies and outcome measures, further research is needed to determine the effectiveness of video game intervention on activity outcomes in children with DCD.

Participation outcomes were only assessed in two articles [36, 42]. Whilst improvements were shown on two questionnaires for participation after video game intervention [36], no significant difference in any physical activity or sedentary time outcomes measured using accelerometry were found between a 16 week video game intervention phase and a 16 week no intervention phase [42]. It should be noted that playing video games is an indoor activity that may restrict outdoor play, with many benefits of outdoor play previously reported [53, 54]. Despite recent video game technology promoting movement through ‘active’ video games, such as the Nintendo Wii or Microsoft Kinect, video game interventions should ensure that the importance of other outdoor play or physical activities is still emphasised and that interventions are not designed that solely focus on children playing indoors.

Four articles also examined TD children after video game intervention [37, 38, 47, 48]. These articles were conducted by the same research team and provided identical video game interventions to groups of children with DCD and TD children. Different results were shown between cohorts, with children with DCD showing larger improvements [38, 47, 48]. It should be noted that TD children would be expected to have higher levels of motor function at baseline and their capacity to improve would not be as large as children with DCD. This is compounded when some items from the MABC-2 and BOT-2 have strong ceiling effects that limit improvement [48]. Two of these studies from the same intervention and cohort also compared variable and repetitive video game training [37, 38]. No significant differences were found on outcomes related to body structure and function or activity between the variable and repetitive video game training [38]. Repetitive training showed significant differences compared to variable training for improvement in video game performance [37], although this is to be expected when the repetitive group trained with the specific video game used for assessment.

Interestingly, this systematic review only identified studies that used video games for intervention, such as the Nintendo Wii. Due to heterogeneity between studies, it was difficult to compare results based on the specific video game device used. Our finding of limited and mixed evidence for video game interventions in paediatric populations is consistent with other reviews of the literature [15]. We did not find any studies that used ‘immersive’ virtual reality technology. Review studies have shown promise for virtual reality technology to potentially improve upper limb function in children with cerebral palsy [16, 21], although research is still limited. Virtual reality technology is still in its infancy, with refinement and development of virtual reality equipment constantly being undertaken. As the video gaming equipment that was used in the studies included in the current review are no longer being manufactured, future research may wish to examine the effects of virtual reality technology on motor impairments, limitations and restrictions in children with DCD.

Other systematic reviews in children with cerebral palsy report similar results to the current review; that research is limited; however, video game interventions are feasible, enjoyable and potentially effective for improved physical function [15, 17]. Of the studies included in this review, the majority of children adhered to and enjoyed the interventions. A similar recent review suggested that video game interventions may provide motor benefits across paediatric cohorts of cerebral palsy, Down syndrome and DCD, although data on adherence and enjoyment were not reported [25]. Interestingly, the previous review only reported six articles in children with DCD compared to the 15 found in the current review. One excluded study from the current review [55], with inadequate description of the condition, examined motor impairments following Nintendo Wii intervention in children with developmental delay. This article also showed that the intervention was feasible and enjoyable for children, as well as potentially effective for improving motor impairment compared with a control group [55]. A recent study by Howie et al. [56] examined further aspects of feasibility of video game intervention in children with DCD, after two studies (included in this review) found limited benefits of Nintendo Wii intervention [42, 49]. The authors suggest that although children with DCD generally reached the prescribed dosage of video game intervention, the quality of play was inadequate in their home-based intervention [56]. Howie et al. [56] recommend a number of factors that need to be considered when designing video game interventions, including detailed guidance to children, supervision of video game play and introducing new games or levels to keep children engaged.

## Conclusion

In conclusion, we found limited research examining video game based interventions for improving body structure and function impairment, activity limitation and participation restriction in children with DCD. The majority of studies used the Nintendo Wii for intervention, with children appearing to enjoy and adhere to the interventions. The effect of the interventions was mixed with conflicting results shown across studies. The studies varied in research design, had relatively low sample sizes and low to moderate methodological quality. Further high quality research is needed to determine the effectiveness of video game based interventions across ICF-CY domains in children with DCD.

## Additional files

Additional file 1: Search terms used for systematic database search. (PDF 112 kb)

Additional file 2: Modified Downs and Black scale with guidelines used for this review. (PDF 131 kb)

Additional file 3: Results of the quality assessment. (PDF 56 kb)

## Abbreviations

BOT2: Bruininks Oseretsky Test of Motor Proficiency, second edition
CSAPPA: Children’s Self-Perceptions of Adequacy in and Predilection for Physical Activity
DCD: Developmental coordination disorder
DCD-Q: Developmental Coordination Disorder Questionnaire
DSM-5: Diagnostic and Statistical Manual for Mental Disorders, fifth edition
ES: Effect size
ICF-CY: International Classification of Functioning, Disability and Health, children and youth version
MABC-2: Movement Assessment Battery for Children, second edition
PADLA-Q: Participation in Activities of Daily Living for Adolescents Questionnaire
PRISMA: Preferred reporting items for systematic reviews and meta-analyses
RCT: Randomised controlled trial
TD: Typically developing

## References

[1] Blank R, Smits-Engelsman B, Polatajko H, Wilson P (2012). European academy for childhood disability (EACD): recommendations on the definition, diagnosis and intervention of developmental coordination disorder (long version). Dev Med Child Neurol.

[2] American Psychiatric Association (2013). Diagnostic and statistical manual of mental disorders.

[3] Wilson PH, Ruddock S, Smits-Engelsman B, Polatajko H, Blank R (2013). Understanding performance deficits in developmental coordination disorder: a meta-analysis of recent research. Dev Med Child Neurol.

[4] Zwicker JG, Harris SR, Klassen AF (2013). Quality of life domains affected in children with developmental coordination disorder: a systematic review. Child Care Health Dev.

[5] Zwicker JG, Suto M, Harris SR, Vlasakova N, Missiuna C (2018). Developmental coordination disorder is more than a motor problem: children describe the impact of daily struggles on their quality of life. Br J Occup Ther.

[6] Cousins M, Smyth MM (2003). Developmental coordination impairments in adulthood. Hum Mov Sci.

[7] Du W, Wilmut K, Barnett AL (2015). Level walking in adults with and without developmental coordination disorder: an analysis of movement variability. Hum Mov Sci.

[8] Hill EL, Brown D (2013). Mood impairments in adults previously diagnosed with developmental coordination disorder. J Ment Health.

[9] Cantell MH, Smyth MM, Ahonen TP (2003). Two distinct pathways for developmental coordination disorder: persistence and resolution. Hum Mov Sci.

[10] Smits-Engelsman BCM, Blank R, Van Der Kaay AC, Mosterd-Van Der Meijs R, Vlugt-van den brand E, Polatajko HJ, Wilson PH (2013). Efficacy of interventions to improve motor performance in children with developmental coordination disorder: a combined systematic review and meta-analysis. Dev Med Child Neurol.

[11] Miyahara M, Hillier SL, Pridham L, Nakagawa S. Task‐oriented interventions for children with developmental co‐ordination disorder. Cochrane Database Syst Rev. 2017;2017(7):CD010914. 10.1002/14651858.CD010914.pub2 PMC6483344 28758189

[12] Preston N, Magallón S, Hill LJB, Andrews E, Ahern SM, Mon-Williams M (2017). A systematic review of high quality randomized controlled trials investigating motor skill programmes for children with developmental coordination disorder. Clin Rehabil.

[13] Smits-Engelsman B, Vinçon S, Blank R, Quadrado VH, Polatajko H, Wilson PH (2018). Evaluating the evidence for motor-based interventions in developmental coordination disorder: a systematic review and meta-analysis. Res Dev Disabil.

[14] Yu JJ, Burnett AF, Sit CH. Motor skill interventions in children with developmental coordination disorder: a systematic review and meta-analysis. Arch Phys Med Rehabil. 2018;99(10):2076-2099. 10.1016/j.apmr.2017.12.009 29329670

[15] Bonnechère B, Jansen B, Omelina L, Degelaen M, Wermenbol V, Rooze M, Van Sint Jan S (2014). Can serious games be incorporated with conventional treatment of children with cerebral palsy?. A review Res Dev Disabil.

[16] Chen YP, Lee SY, Howard AM (2014). Effect of virtual reality on upper extremity function in children with cerebral palsy: a meta-analysis. Pediatr Phys Ther.

[17] Mitchell L, Ziviani J, Oftedal S, Boyd R (2012). The effect of virtual reality interventions on physical activity in children and adolescents with early brain injuries including cerebral palsy. Dev Med Child Neurol.

[18] Sandlund M, McDonough S, Häger-Ross C (2009). Interactive computer play in rehabilitation of children with sensorimotor disorders: a systematic review. Dev Med Child Neurol.

[19] LeBlanc AG, Chaput JP, McFarlane A, Colley RC, Thivel D, Biddle SJH, Maddison R, Leatherdale ST, Tremblay MS (2013). Active video games and health indicators in children and youth: a systematic review. PLoS One.

[20] Parsons TD, Rizzo AA, Rogers S, York P (2009). Virtual reality in paediatric rehabilitation: a review. Dev Neurorehabil..

[21] Galvin J, McDonald R, Catroppa C, Anderson V (2011). Does intervention using virtual reality improve upper limb function in children with neurological impairment: a systematic review of the evidence. Brain Inj.

[22] Laufer Y, Weiss PL (2011). Virtual reality in the assessment and treatment of children with motor impairment: a systematic review. J Phys Ther Educ.

[23] Snider L, Majnemer A, Darsaklis V (2010). Virtual reality as a therapeutic modality for children with cerebral palsy. Dev Neurorehabil.

[24] Weiss PL, Tirosh E, Fehlings D (2014). Role of virtual reality for cerebral palsy management. J Child Neurol.

[25] Hickman R, Popescu L, Manzanares R, Morris B, Lee S-P, Dufek JS (2017). Use of active video gaming in children with neuromotor dysfunction: a systematic review. Dev Med Child Neurol.

[26] Moher D, Liberati A, Tetzlaff J, Altman DG, Altman D, Antes G, Atkins D, Barbour V, Barrowman N, Berlin JA (2009). Preferred reporting items for systematic reviews and meta-analyses: the PRISMA statement. PLoS Med.

[27] World Health Organisation (2007). International classification of functioning, disability and health: children and youth version (ICF-CY).

[28] Randall M, Johnson L, Reddihough D (1999). The Melbourne assessment of unilateral upper limb function: test administration manual.

[29] Downs SH, Black N (1998). The feasibility of creating a checklist for the assessment of the methodological quality both of randomised and non-randomised studies of health care interventions. J Epidemiol Community Health.

[30] Samoocha D, Bruinvels DJ, Elbers NA, Anema JR, van der Beek AJ (2010). Effectiveness of web-based interventions on patient empowerment: a systematic review and meta-analysis. J Med Internet Res.

[31] Simic M, Hinman RS, Wrigley TV, Bennell KL, Hunt MA (2011). Gait modification strategies for altering medial knee joint load: a systematic review. Arthritis Care Res.

[32] Searle A, Spink M, Ho A, Chuter V (2015). Exercise interventions for the treatment of chronic low back pain: a systematic review and meta-analysis of randomised controlled trials. Clin Rehabil.

[33] Munn J, Sullivan SJ, Schneiders AG (2010). Evidence of sensorimotor deficits in functional ankle instability: a systematic review with meta-analysis. J Sci Med Sport.

[34] Darrah J, Hickman R, O'Donnell M, Vogtle L, Wiart L. AACPDM methodology to develop systematic reviews of treatment interventions (Revision 1.2). 2008. www.aacpdm.org. Accessed 13 Sept 2016.

[35] Ashkenazi T, Weiss PL, Orian D, Laufer Y (2013). Low-cost virtual reality intervention program for children with developmental coordination disorder: a pilot feasibility study. Pediatr Phys Ther.

[36] Bonney E, Ferguson G, Smits-Engelsman B (2017). The efficacy of two activity-based interventions in adolescents with developmental coordination disorder. Res Dev Disabil.

[37] Bonney E, Jelsma D, Ferguson G, Smits-Engelsman B (2017). Variable training does not lead to better motor learning compared to repetitive training in children with and without DCD when exposed to active video games. Res Dev Disabil.

[38] Bonney E, Jelsma LD, Ferguson GD, Smits-Engelsman BCM (2017). Learning better by repetition or variation? Is transfer at odds with task specific training?. PLoS One.

[39] Bonney E, Rameckers E, Ferguson G, Smits-Engelsman B (2018). "Not just another Wii training": A graded Wii protocol to increase physical fitness in adolescent girls with probable developmental coordination disorder-a pilot study. BMC Pediatr.

[40] Ferguson GD, Jelsma D, Jelsma J, Smits-Engelsman BCM (2013). The efficacy of two task-orientated interventions for children with developmental coordination disorder: Neuromotor task training and Nintendo Wii fit training. Res Dev Disabil.

[41] Hammond J, Jones V, Hill EL, Green D, Male I (2014). An investigation of the impact of regular use of the Wii fit to improve motor and psychosocial outcomes in children with movement difficulties: a pilot study. Child Care Health Dev.

[42] Howie EK, Campbell AC, Straker LM (2016). An active video game intervention does not improve physical activity and sedentary time of children at-risk for developmental coordination disorder: a crossover randomized trial. Child Care Health Dev.

[43] Jelsma D, Geuze RH, Mombarg R, Smits-Engelsman BCM (2014). The impact of Wii fit intervention on dynamic balance control in children with probable developmental coordination disorder and balance problems. Hum Mov Sci.

[44] Jelsma LD, Smits-Engelsman BCM, Krijnen WP, Geuze RH (2016). Changes in dynamic balance control over time in children with and without developmental coordination disorder. Hum Mov Sci.

[45] Ju YJ, Du YC, Lin LY, Hou CR, Lin PY, Cherng RJ. The effect of laboratory-developed video games on balance performance in children with developmental coordination disorder. Biomed Eng Appl Basis Commun. 2018;30(1).

[46] Mombarg R, Jelsma D, Hartman E (2013). Effect of Wii-intervention on balance of children with poor motor performance. Res Dev Disabil.

[47] Smits-Engelsman BCM, Jelsma LD, Ferguson GD (2016). The effect of exergames on functional strength, anaerobic fitness, balance and agility in children with and without motor coordination difficulties living in low-income communities. Hum Mov Sci.

[48] Smits-Engelsman BCM, Jelsma LD, Ferguson GD, Geuze RH (2015). Motor learning: an analysis of 100 trials of a ski slalom game in children with and without developmental coordination disorder. PLoS One.

[49] Straker L, Howie E, Smith A, Jensen L, Piek J, Campbell A (2015). A crossover randomised and controlled trial of the impact of active video games on motor coordination and perceptions of physical ability in children at risk of developmental coordination disorder. Hum Mov Sci.

[50] Aertssen WFM, Ferguson GD, Smits-Engelsman BCM (2016). Reliability and structural and construct validity of the functional strength measurement in children aged 4 to 10 years. Phys Ther.

[51] Verschuren O, Takken T, Ketelaar M, Gorter JW, Helders PJM (2007). Reliability for running tests for measuring agility and anaerobic muscle power in children and adolescents with cerebal palsy. Pediatr Phys Ther.

[52] Green D, Wilson BN (2008). The importance of parent and child opinion in detecting change in movement capabilities. Can J Occup Ther.

[53] Fjørtoft I (2001). The natural environment as a playground for children: the impact of outdoor play activities in pre-primary school children. Early Child Educ J.

[54] Mainella FP, Agate JR, Clark BS (2011). Outdoor-based play and reconnection to nature: a neglected pathway to positive youth development. New Dir Youth Dev.

[55] Salem Y, Gropack SJ, Coffin D, Godwin EM (2012). Effectiveness of a low-cost virtual reality system for children with developmental delay: a preliminary randomised single-blind controlled trial. Physiotherapy.

[56] Howie EK, Campbell AC, Abbott RA, Straker LM (2017). Understanding why an active video game intervention did not improve motor skill and physical activity in children with developmental coordination disorder: a quantity or quality issue?. Res Dev Disabil.

