# Supplementary material for: Do video game interventions improve motor outcomes in children with developmental coordination disorder? A systematic review using the ICF framework
Source: BMC Pediatr. 2019 Jan 16;19:22. doi: 10.1186/s12887-018-1381-7 (PMC6335818; doi:10.1186/s12887-018-1381-7)
Supplement: Supplementary file 1 — Search terms used for systematic database search. (PDF 112 kb) [file 12887_2018_1381_MOESM1_ESM.pdf]

Additional file 1. Search terms used for systematic database search.

	Search terms
**Population**	
Children	paediatric* OR pediatric* OR child* OR adolesc*
Neurodevelopmental conditions	Developmental OR neurodevelopmental OR "cerebral palsy" OR "Down's syndrome" OR “Downs syndrome” OR "Down syndrome" OR preterm OR pre-term OR premature OR "muscular dystrophy" OR autism OR "spina bifida" OR “brain injur*”
**Intervention**	
Virtual reality	"virtual realit*" OR "virtual therap*" OR "virtual environment*" OR "video gam*" OR "active gam*" OR exergam* OR "computer gam*" OR "serious gam*" OR Wii OR Kinect OR PlayStation OR EyeToy OR GestureTek OR IREX
**Comparison**	
N/A	
**Outcome**	
Physical function or motor impairment	walk* OR balance OR equilibrium OR postur* OR "body sway" OR "lower limb" OR “lower extremit*” OR stand* OR step* OR ambulat* OR motor OR fitness OR “cardiovascular endurance” OR activit* OR exercis* OR sedentary OR locomot* OR energy

Each domain searched independently, with domains combined using ‘AND’. Relevant medical subject headings were also included in applicable databases. A variety of neurodevelopmental conditions were included to ensure a comprehensive search of the available literature was performed to identify any study that included children with developmental coordination disorder.
