# Supplementary material for: Do video game interventions improve motor outcomes in children with developmental coordination disorder? A systematic review using the ICF framework
Source: BMC Pediatr. 2019 Jan 16;19:22. doi: 10.1186/s12887-018-1381-7 (PMC6335818; doi:10.1186/s12887-018-1381-7)
Supplement: Supplementary file 2 — Modified Downs and Black scale with guidelines used for this review. (PDF 131 kb) [file 12887_2018_1381_MOESM2_ESM.pdf]

Additional file 2. Modified Downs and Black scale with guidelines used for this review.

Questions	Guidelines
Reporting	
1. Is the hypothesis/aim/objective of the study clearly described?	**Yes**: includes clear description of either aim or hypothesis<br>**No**: does not include clear description of either aim or hypothesis
2. Are the main outcomes to be measured clearly described in the Introduction or Methods section?<br>If the main outcomes are first mentioned in the Results section, the question should be answered no.	**Yes**: outcomes clearly described in appropriate section<br>**No**: outcomes not clearly described or first mentioned in the Results section
3. Are the characteristics of the patients included in the study clearly described?	**Yes**: clear description of the DCD cohort including selection criteria (even if control group is unclearly described)<br>**No**: unclear description or no selection criteria for DCD cohort
4. Are the interventions of interest clearly described?<br>Treatments and placebo (where relevant) that are to be compared should be clearly described.	**Yes**: clearly described and potential to be reproduced<br>**No**: not clearly described and difficult to be reproduced
5. Are the distributions of principal confounders in each group of subjects to be compared clearly described?	**Yes**: age and gender identified<br>**Partially**: age or gender identified<br>**No**: neither age or gender identified
6. Are the main findings of the study clearly described?<br>Simple outcome data (including denominators and numerators) should be reported for all major findings so that the reader can check the major analyses and conclusions. (This question does not cover statistical tests which are considered below).	**Yes**: complete description including pre/post scores<br>**No**: incomplete description e.g. with just change scores
7. Does the study provide estimates of the random variability in the data for the main outcomes?<br>In non-normally distributed data the inter-quartile range of results should be reported. In normally distributed data the standard error, standard deviation or confidence intervals should be reported. If the distribution of the data is not described, it must be assumed that the estimates used were appropriate and the question should be answered yes.	**Yes**: provides standard deviation or inter-quartile range or measure of variability for main outcome measures<br>**No**: no measure of variability (e.g. only mean)
8. Have all important adverse events that may be a consequence of the intervention been reported?<br>This should be answered yes if the study demonstrates that there was a comprehensive attempt to measure adverse events (a list of possible adverse events is provided).	**Yes**: measured or described adverse events even if there were none<br>**No**: no attempt to measure or describe adverse events

9. Have the characteristics of patients lost to follow-up been described?<br>This should be answered yes where there were no losses to follow-up or where losses to follow-up were so small that findings would be unaffected by their inclusion. This should be no, where a study does not report the number of patients lost to follow-up.	**Yes**: if there is no loss to follow-up; or if follow-up is greater than or equal to 85%; or if follow-up is 75-85% but patients lost to follow-up have been described<br>**No**: less than 85% follow-up and no description of lost patients; less than 75% follow up
10. Have actual probability values been reported (e.g. 0.035 rather than <0.05) for the main outcomes except where the probability value is less than 0.001?	**Yes**: *p*-value reported with decimal places (e.g. 0.031)<br>**No**: *p*-value reported as <0.05
External validity	
11. Were the subjects asked to participate in the study representative of the entire population from which they were recruited?<br>The study must identify the source population for patients and describe how the patients were selected. Patients would be representative if they comprised the entire source population, an unselected sample of consecutive patients, or a random sample. Random sampling is only feasible where a list of all members of the relevant population exists. Where a study does not report the proportion of the source population from which the patients are derived, the question should be answered as unable to determine.	**Yes**: population study; consecutively or randomly sampled<br>**UTD**: no description of proportion of population or sampling methods<br>**No**: states that it is a convenience sample or methods clearly not representative of entire population
12. Were those subjects who were prepared to participate representative of the entire population from which they were recruited?<br>The proportion of those asked who agreed should be stated. Validation that the sample was representative would include demonstrating that the distribution of the main confounding factors was the same in the study sample and the source population.	**Yes**: provides a comparison of study sample to overall population<br>**UTD**: if they score UTD for the question before; if they do not state how many were asked and how many agreed<br>**No**: if they score no for the question prior
13. Were the staff, places, and facilities where the patients were treated, representative of the treatment the majority of patients receive?<br>For the question to be answered yes the study should demonstrate that the intervention was representative of that in use in the source population. The question should be answered no if, for example, the intervention was undertaken in a specialist centre unrepresentative of the hospitals most of the source population would attend.	**Yes**: intervention performed in a school or at home<br>**UTD**: not mentioned where intervention is performed<br>**No**: intervention performed in a lab setting; inpatient hospital setting
Internal validity - bias	
14. Was an attempt made to blind study subjects to the intervention they have received?<br>For studies where the patients would have no way of knowing which intervention they received, this should be answered yes.	**Yes**: participants do not know if receiving experimental intervention or control<br>**UTD**: no description of intervention protocol or no description of blinding study subjects<br>**No**: if only one group; if crossover design with actual control group that does nothing

15. Was an attempt made to blind those measuring the main outcomes of the intervention?	**Yes**: clearly state assessors were blinded<br>**UTD**: no description of assessors<br>**No**: describe assessors but no mention of blinding; describe assessors and state they were not blinded
16. If any of the results of the study were based on "data dredging", was this made clear?<br>Any analyses that had not been planned at the outset of the study should be clearly indicated. If no retrospective unplanned subgroup analyses were reported, then answer yes.	**Yes**: if they followed the aims and only did analyses related to aims; additional analyses but clearly stated that it was a secondary analysis<br>**UTD**: no description of aims<br>**No**: did unplanned analyses that were not related to the aims without mention of why
17. In trials and cohort studies, do the analyses adjust for different lengths of follow-up of patients, or in case-control studies, is the time period between the intervention and outcome the same for cases and controls?<br>Where follow-up was the same for all study patients the answer should yes. If different lengths of follow-up were adjusted for by, for example, survival analysis the answer should be yes. Studies where differences in follow-up are ignored should be answered no.	**Yes**: same period of time for all groups between last training and post-assessment<br>**UTD**: no mention of time period between last training and post-assessment<br>**No**: different time periods between last training sessions and post-assessment without adjustment
18. Were the statistical tests used to assess the main outcomes appropriate?<br>The statistical techniques used must be appropriate to the data. For example, nonparametric methods should be used for small sample sizes. Where little statistical analysis has been undertaken but where there is no evidence of bias, the question should be answered yes. If the distribution of the data (normal or not) is not described it must be assumed that the estimates used were appropriate and the question should be answered yes.	**Yes**: appropriate statistical methods<br>**UTD**: statistical methods not reported<br>**No**: obvious error in statistical methods (e.g. using parametric methods for non-normally distributed data)
19. Was compliance with the intervention/s reliable?<br>Where there was noncompliance with the allocated treatment or where there was contamination of one group, the question should be answered no. For studies where the effect of any misclassification was likely to bias any association to the null, the question should be answered yes.	**Yes**: the study states ways to avoid contamination or reports compliance/adherence (greater than or equal to 85%)<br>**UTD**: no mention of compliance/adherence and contamination<br>**No**: State that the non-video game kids played video games outside of the intervention; intervention included other aspects that were not video game related; less than 85% adherence/compliance
20. Were the main outcome measures used accurate (valid and reliable)?<br>For studies where the outcome measures are clearly described, the question should be answered yes. For studies which refer to other work or that demonstrates the outcome measures are accurate, the question should be answered as yes.	**Yes**: if the study measured reliability or validity or if the study referenced prior work (for the majority of outcomes)<br>**UTD**: if the study used a commonly used assessment (e.g. MABC-2, DCD-Q) but no mention of reliability or validity or no mention of previous research<br>**No**: use non-standardised assessments with no mention of reliability/validity statistics

External validity - confounding	
21. Were the patients in different intervention groups (trials and cohort studies) or were the cases and controls (case-control studies) recruited from the same population? For example, patients for all comparison groups should be selected from the same hospital. The question should be answered unable to determine for cohort and case-control studies where there is no information concerning the source of patients included in the study.	**Yes**: all participants recruited from the same school, community, or hospital<br>**UTD**: no information where participants were recruited<br>**No**: participants recruited from different schools, communities in different areas (e.g. rural vs city)
22. Were study subjects in different intervention groups (trials and cohort studies) or were the cases and controls (case-control studies) recruited over the same period of time? For a study which does not specify the time period over which patients were recruited, the question should be answered as unable to determine.	**Yes**: specifies time period of recruitment<br>**UTD**: no mention of time period of recruitment<br>**No**: time periods that were very different between groups
23. Were study subjects randomised to intervention groups? Studies which state that subjects were randomised should be answered yes except where method of randomisation would not ensure random allocation. For example, alternate allocation would score no because it is predictable.	**Yes**: clearly states randomisation was performed<br>**UTD**: no mention of randomisation<br>**No**: not randomised
24. Was the randomised intervention assignment concealed from both patients and health care staff until recruitment was complete and irrevocable? All non-randomised studies should be answered no. If assignment was concealed from patients but not from staff, it should be answered no.	**Yes**: mention of concealment for both assessors and patients<br>**UTD**: no mention of concealment<br>**No**: non-randomised studies; concealed from patients not assessors or vice versa; states that assignment was not concealed
25. Was there adequate adjustment for confounding in the analyses from which the main findings were drawn? This question should be answered no for trials if: the main conclusions of the study were based on analyses of treatment rather than intention to treat; the distribution of known confounders in the different treatment groups was not described; or the distribution of known confounders differed between the treatment groups but was not taken into account in the analyses. In nonrandomised studies if the effect of the main confounders was not investigated or confounding was demonstrated but no adjustment was made in the final analyses the question should be answered as no.	**Yes**: if studies matched in design for age or gender; adjusted for age or gender in analyses<br>**UTD**: no mention of matching or adjusting in analyses<br>**No**: not matched in design for age or gender and no adjustment in analyses
26. Were losses of patients to follow-up taken into account? If the numbers of patients lost to follow-up are not reported, the question should be answered as unable to determine. If the proportion lost to follow-up was too small to affect the main findings, the question should be answered yes.	**Yes**: if the study reports losses and it is greater than or equal to 85%; study reports no loss to follow-up<br>**UTD**: no mention of losses to follow-up<br>**No**: less than 85% loss to follow-up

Power	
27. Did the study report a sample size or power calculation?	**Yes**: study clearly states sample size calculation<br>**No**: no mention of sample size calculation

Yes, 1 point; Partially, 0.5 points; No, 0 points; UTD, unable to determine (0 points); DCD, developmental coordination disorder; MABC-2, Movement Assessment Battery for Children, second edition; DCD-Q, Developmental Coordination Disorder Questionnaire.
