# Supplementary material for: Do video game interventions improve motor outcomes in children with developmental coordination disorder? A systematic review using the ICF framework
Source: BMC Pediatr. 2019 Jan 16;19:22. doi: 10.1186/s12887-018-1381-7 (PMC6335818; doi:10.1186/s12887-018-1381-7)
Supplement: Supplementary file 3 — Results of the quality assessment. (PDF 56 kb) [file 12887_2018_1381_MOESM3_ESM.pdf]

Additional file 3. Results of the quality assessment.

Author	Question																											Total Score
	**1**	**2**	**3**	**4**	**5**	**6**	**7**	**8**	**9**	**10**	**11**	**12**	**13**	**14**	**15**	**16**	**17**	**18**	**19**	**20**	**21**	**22**	**23**	**24**	**25**	**26**	**27**	
Ashkenazi et al [35]	Y	Y	Y	Y	Y	Y	Y	N	Y	Y	N	N	U	N	Y	Y	Y	Y	N	Y	Y	U	N	N	U	Y	N	16/27 (59%)
Bonney et al [36]	Y	Y	Y	Y	Y	Y	Y	N	Y	Y	N	N	Y	N	Y	Y	Y	Y	Y	Y	Y	U	Y	N	N	Y	Y	20/27 (74%)
Bonney et al [37]	Y	Y	Y	Y	Y	Y	Y	N	Y	Y	N	N	Y	U	Y	Y	Y	Y	Y	Y	Y	U	Y	U	Y	Y	Y	21/27 (78%)
Bonney et al [38]	Y	Y	Y	Y	Y	Y	Y	N	Y	Y	N	N	Y	U	Y	Y	Y	Y	Y	N	Y	U	Y	U	Y	Y	Y	20/27 (74%)
Bonney et al [39]	Y	Y	Y	Y	Y	Y	Y	Y	Y	Y	N	N	Y	N	Y	Y	U	Y	Y	Y	Y	U	N	N	N	Y	Y	19/27 (70%)
Ferguson et al [40]	Y	Y	Y	Y	Y	Y	Y	N	Y	Y	N	N	Y	U	Y	Y	N	Y	Y	Y	Y	U	N	N	U	Y	Y	18/27 (67%)
Hammond et al [41]	Y	Y	Y	N	Y	Y	Y	N	Y	Y	N	N	Y	N	N	Y	Y	Y	Y	Y	Y	U	Y	U	U	Y	N	17/27 (63%)
Howie et al [42]	Y	Y	Y	Y	Y	Y	Y	N	Y	Y	N	N	Y	N	U	Y	Y	Y	Y	N	U	Y	Y	N	U	Y	Y	18/27 (67%)
Jelsma et al [43]	Y	Y	Y	Y	Y	Y	Y	N	Y	Y	N	N	Y	N	N	Y	U	Y	Y	Y	N	U	U	U	U	Y	N	15/27 (56%)
Jelsma et al [44]	Y	Y	Y	Y	Y	Y	Y	N	Y	Y	N	N	Y	N	N	Y	U	Y	Y	N	N	U	U	U	Y	Y	N	15/27 (56%)
Ju et al [45]	Y	Y	Y	N	Y	Y	Y	N	Y	N	N	N	Y	N	U	Y	U	Y	U	N	Y	U	Y	U	Y	Y	N	14/27 (52%)
Mombarg et al [46]	Y	Y	Y	Y	Y	Y	Y	N	Y	Y	N	N	Y	N	U	Y	U	Y	Y	Y	Y	U	Y	U	U	Y	N	17/27 (63%)
Smits-Engelsman et al [47]	Y	Y	Y	Y	Y	Y	Y	N	Y	Y	N	N	Y	N	U	Y	U	Y	Y	Y	Y	U	N	N	Y	Y	N	17/27 (63%)
Smits-Engelsman et al [48]	Y	Y	Y	Y	Y	Y	Y	N	Y	Y	N	N	Y	N	Y	Y	U	Y	Y	Y	Y	U	N	N	Y	Y	N	18/27 (67%)
Straker et al [49]	Y	Y	Y	Y	Y	N	Y	N	Y	Y	N	N	Y	N	U	Y	Y	Y	Y	Y	U	Y	Y	N	U	Y	Y	18/27 (67%)

Y, yes (1 point); N, no (0 points); U, unable to determine (0 points). See Additional file 2 for detailed description of each question. Data retrieved from each article, related articles or by contacting authors.

References for Additional file 3

35. Ashkenazi T, Weiss PL, Orian D, Laufer Y. Low-cost virtual reality intervention program for children with developmental coordination disorder: A pilot feasibility study. Pediatr Phys Ther. 2013;25(4):467-473.

36. Bonney E, Ferguson G, Smits-Engelsman B. The efficacy of two activity-based interventions in adolescents with developmental coordination disorder. Res Dev Disabil. 2017a;71:223-236.

37. Bonney E, Jelsma D, Ferguson G, Smits-Engelsman B. Variable training does not lead to better motor learning compared to repetitive training in children with and without DCD when exposed to active video games. Res Dev Disabil. 2017b;62:124-136.

38. Bonney E, Jelsma LD, Ferguson GD, Smits-Engelsman BCM. Learning better by repetition or variation? Is transfer at odds with task specific training? PLoS ONE. 2017c;12(3):e0174214.

39. Bonney E, Rameckers E, Ferguson G, Smits-Engelsman B. "Not just another Wii training": A graded Wii protocol to increase physical fitness in adolescent girls with probable developmental coordination disorder-a pilot study. BMC Pediatr. 2018;18(1):78.

40. Ferguson GD, Jelsma D, Jelsma J, Smits-Engelsman BCM. The efficacy of two task-orientated interventions for children with developmental coordination disorder: Neuromotor task training and Nintendo Wii Fit training. Res Dev Disabil. 2013;34(9):2449-2461.

41. Hammond J, Jones V, Hill EL, Green D, Male I. An investigation of the impact of regular use of the Wii Fit to improve motor and psychosocial outcomes in children with movement difficulties: A pilot study. Child Care Health Dev. 2014;40(2):165-175.

42. Howie EK, Campbell AC, Straker LM. An active video game intervention does not improve physical activity and sedentary time of children at-risk for developmental coordination disorder: A crossover randomized trial. Child Care Health Dev. 2016;42(2):253-260.

43. Jelsma D, Geuze RH, Mombarg R, Smits-Engelsman BCM. The impact of Wii Fit intervention on dynamic balance control in children with probable developmental coordination disorder and balance problems. Hum Mov Sci. 2014;33(1):404-418.

44. Jelsma LD, Smits-Engelsman BCM, Krijnen WP, Geuze RH. Changes in dynamic balance control over time in children with and without developmental coordination disorder. Hum Mov Sci. 2016;49:148-159.

45. Ju YJ, Du YC, Lin LY, Hou CR, Lin PY, Cherng RJ. The effect of laboratory-developed video games on balance performance in children with developmental coordination disorder. Biomed Eng Appl Basis Commun. 2018;30(1).

46. Mombarg R, Jelsma D, Hartman E. Effect of Wii-intervention on balance of children with poor motor performance. Res Dev Disabil. 2013;34(9):2996-3003.

47. Smits-Engelsman BCM, Jelsma LD, Ferguson GD. The effect of exergames on functional strength, anaerobic fitness, balance and agility in children with and without motor coordination difficulties living in low-income communities. Hum Mov Sci. 2016;55:327-337.

48. Smits-Engelsman BCM, Jelsma LD, Ferguson GD, Geuze RH. Motor learning: An analysis of 100 trials of a ski slalom game in children with and without developmental coordination disorder. PLoS ONE. 2015;10(10):e0140470.

49. Straker L, Howie E, Smith A, Jensen L, Piek J, Campbell A. A crossover randomised and controlled trial of the impact of active video games on motor coordination and perceptions of physical ability in children at risk of developmental coordination disorder. Hum Mov Sci. 2015;42:146-160.
